# Supplementary figures and images for: #MaskOn! #MaskOff! Digital polarization of mask-wearing in the United States during COVID-19
Source: PLoS One. 2021 Apr 28;16(4):e0250817. doi: 10.1371/journal.pone.0250817 (PMC8081244; doi:10.1371/journal.pone.0250817)

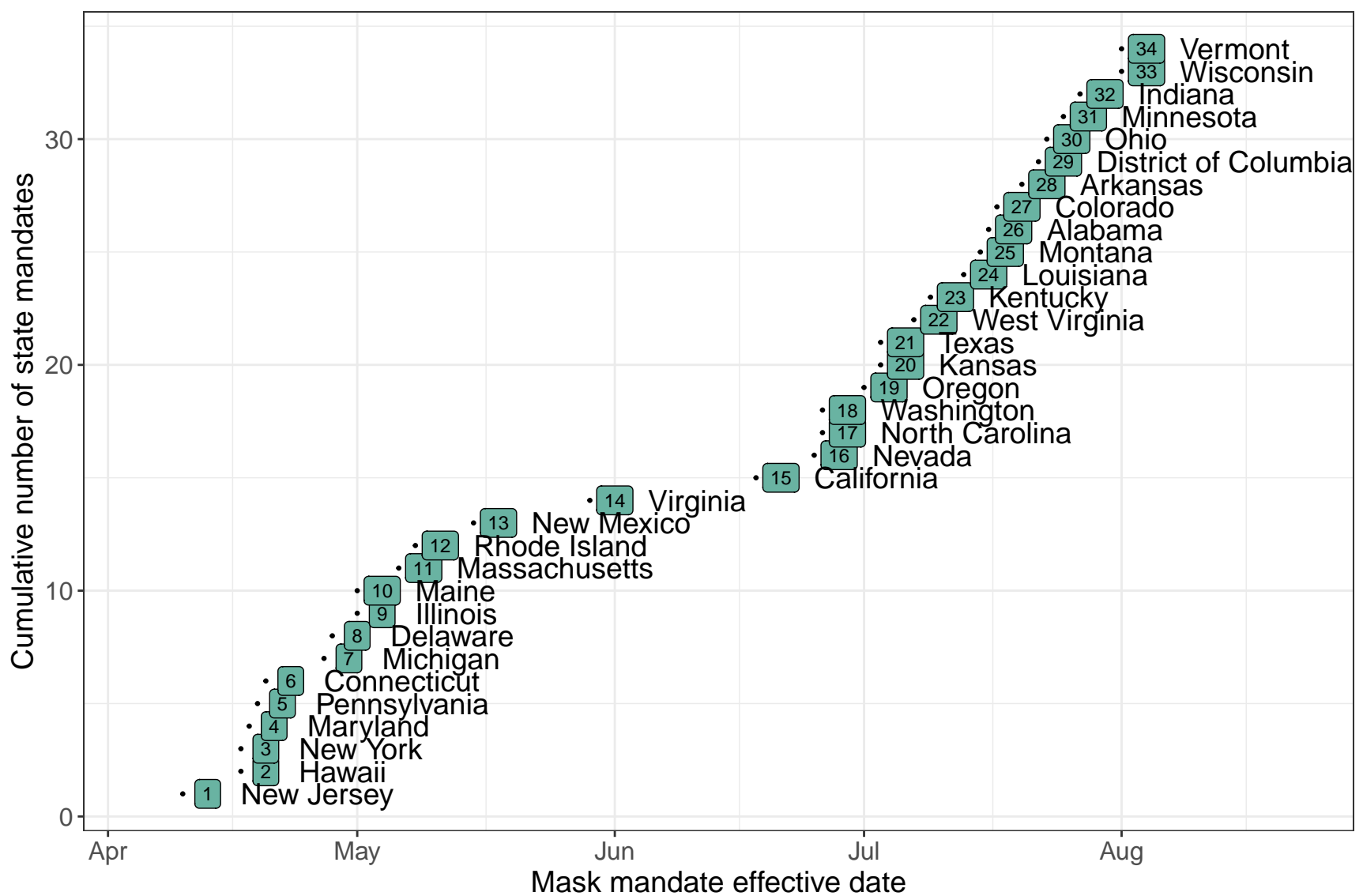

Supplement: S1 Fig — The figure shows the effective dates of executive mask mandates (33 states plus D.C.) on the x-axis and the cumulative number of mandates on the y-axis over the time period under study. (PDF) [file pone.0250817.s002.pdf]

Google Trends searches for *face mask* (2020)

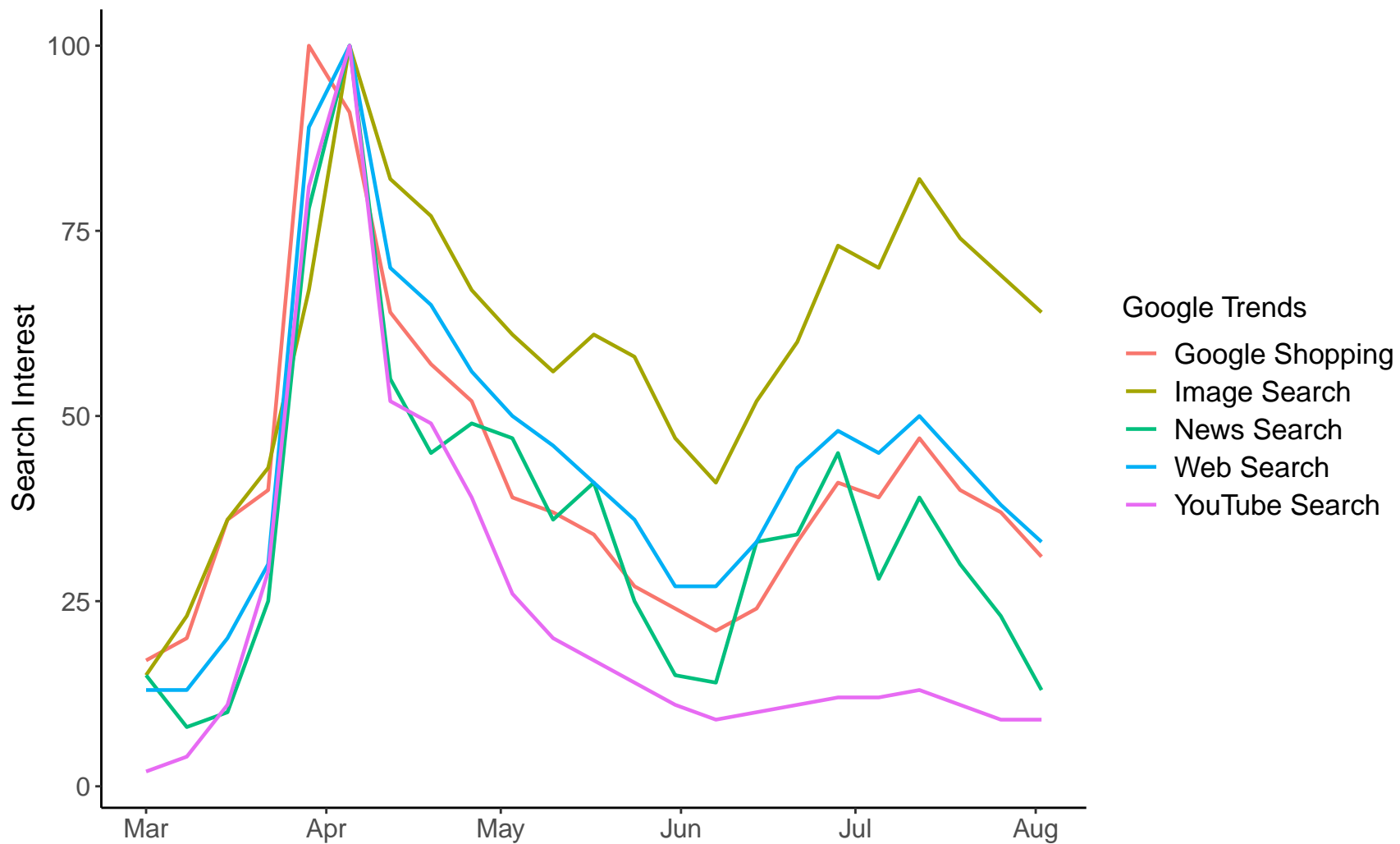

Supplement: S2 Fig — The figure shows the trajectories of weekly search interests for face mask on Google Trends in the U.S. over the time period under study. The vertical axis represents the relative interest to total search volume. Searches for face mask spiked in early April in all five search categories, coinciding with the CDC mask recommendation. After the sharp decline, search interests rebounded to a second peak in late June and increased further in mid-July in most categories except YouTube search. (PDF) [file pone.0250817.s003.pdf]
