# Supplementary material for: #MaskOn! #MaskOff! Digital polarization of mask-wearing in the United States during COVID-19
Source: PLoS One. 2021 Apr 28;16(4):e0250817. doi: 10.1371/journal.pone.0250817 (PMC8081244; doi:10.1371/journal.pone.0250817)
Supplement: S1 Table — (PDF) [file pone.0250817.s004.pdf]

S1 Table. Chi-square test results of independence for six trending issues and baseline (data from Knight Foundation 2019)

Issues	Segment	Percent	Chi-square	df	p-value
Baseline	Extreme	35			
	Moderate	65			
Hurricane	Extreme	18.5	6.702	1	0.010*
	Moderate	81.5			
North Korea	Extreme	22	4.147	1	0.042*
	Moderate	78			
Mueller Investigation	Extreme	34	0.089	1	0.765
	Moderate	66			
Sexual Harassment	Extreme	26	1.911	1	0.167
	Moderate	74			
White nationalism	Extreme	20	5.643	1	0.018*
	Moderate	80			
Mass shootings	Extreme	30	0.570	1	0.450
	Moderate	70			

*$p < 0.05$. Extreme: Extreme Left and Extreme Right; Moderate: Center Left and Center Right

References

Freelon, Deen. (2019). Tweeting left, right, & center: How users and attention are distributed across Twitter. The John S. and James L. Knight Foundation.
