# Supplementary material for: #MaskOn! #MaskOff! Digital polarization of mask-wearing in the United States during COVID-19
Source: PLoS One. 2021 Apr 28;16(4):e0250817. doi: 10.1371/journal.pone.0250817 (PMC8081244; doi:10.1371/journal.pone.0250817)
Supplement: S2 Table — (PDF) [file pone.0250817.s005.pdf]

S2 Table. Statewide mask mandates (DC included) and effective dates between March and August 2020

State	Effective Date	Order Link
New Jersey	4/10	https://nj.gov/infobank/eo/056murphy/pdf/EO-122.pdf
Hawaii	4/17	https://governor.hawaii.gov/wp-content/uploads/2020/04/2004088-ATG_Fifth-Supplementary-Proclamation-for-COVID-19-distribution-signed.pdf
New York	4/17	https://www.governor.ny.gov/news/no-20218-continuing-temporary-suspension-and- modification-laws-relating-disaster-emergency
Maryland	4/18	https://governor.maryland.gov/wp-content/uploads/2020/04/Masks-and-Physical- Distancing-4.15.20.pdf
Pennsylvania	4/19	https://www.governor.pa.gov/wp-content/uploads/2020/04/20200415-SOH-worker-safety- order.pdf
Connecticut	4/20	https://portal.ct.gov/-/media/Office-of-the-Governor/Executive-Orders/Lamont-Executive- Orders/Executive-Order-No-7BB.pdf?la=en
Michigan	4/27	https://content.govdelivery.com/attachments/MIEOG/2020/04/24/file_attachments/1435194 /EO%202020-59.pdf
Delaware	4/28	https://governor.delaware.gov/health-soe/thirteenth-state-of-emergency/
Illinois	5/1	https://www2.illinois.gov/Pages/Executive-Orders/ExecutiveOrder2020-32.aspx
Maine	5/1	https://www.maine.gov/governor/mills/sites/maine.gov.governor.mills/files/inline- files/An%20Order%20to%20Stay%20Safer%20at%20Home.pdf
Massachusetts	5/6	https://www.mass.gov/doc/may-1-2020-masks-and-face-coverings/download
Rhode Island	5/8	https://governor.ri.gov/documents/orders/Executive-Order-20-30.pdf
New Mexico	5/15	https://cv.nmhealth.org/wp-content/uploads/2020/05/5-15-2020-PHO.pdf
Virginia	5/29	https://www.governor.virginia.gov/media/governorvirginiagov/executive-actions/EO-63-and-Order-Of-Public-Health-Emergency-Five---Requirement-To-Wear-Face-Covering-While-Inside-Buildings.pdf
California	6/18	https://www.cdph.ca.gov/Programs/CID/DCDC/CDPH%20Document%20Library/COVID-19/Guidance-for-Face-Coverings_06-18-2020.pdf
Nevada	6/25	https://nvhealthresponse.nv.gov/wp-content/uploads/2020/06/Directive-024-Face-Coverings.pdf
North Carolina	6/26	https://files.nc.gov/governor/documents/files/EO147-Phase-2-Extension.pdf
Washington	6/26	https://coronavirus.wa.gov/information-for/you-and-your-family/face-masks-or-cloth-face-covering
Oregon	7/1	https://sharedsystems.dhsoha.state.or.us/DHSForms/Served/le2288K.pdf
Kansas	7/3	https://governor.kansas.gov/wp-content/uploads/2020/07/20200702093130003.pdf
Texas	7/3	https://open.texas.gov/uploads/files/organization/opentexas/EO-GA-29-use-of-face-coverings-during-COVID-19-IMAGE-07-02-2020.pdf
West Virginia	7/7	https://governor.wv.gov/Documents/2020%20Executive%20Orders/EO%2050-20.pdf
Kentucky	7/9	https://governor.ky.gov/attachments/20200904_Executive-Order_2020-750_Face-Coverings.pdf
Louisiana	7/13	https://gov.louisiana.gov/assets/Proclamations/2020/89-JBE-2020.pdf
Montana	7/15	https://covid19.mt.gov/Portals/223/Documents/Mask%20Directive%20FINAL.pdf?ver=2020-07-15-140109-633
Alabama	7/16	http://alabamapublichealth.gov/legal/assets/order-adph-cov-gatherings-072920.pdf
Colorado	7/17	https://www.colorado.gov/governor/sites/default/files/inline-files/D 2020 138 Mask Order.pdf
Arkansas	7/20	https://governor.arkansas.gov/images/uploads/executiveOrders/EO_20-43.pdf

District of Columbia	7/22	https://mayor.dc.gov/sites/default/files/dc/sites/mayormb/release_content/attachments/Mayor%27s%20Order%202020-080%20Wearing%20Masks%20in%20DC%20to%20Prevent%20COVID19.pdf
Ohio	7/23	https://governor.ohio.gov/wps/portal/gov/governor/media/news-and-media/covid19-update-07222020
Minnesota	7/25	https://www.leg.mn.gov/archive/execorders/20-81.pdf
Indiana	7/27	https://www.in.gov/gov/files/Executive%20Order%2020-37%20Face%20Covering%20Requirement.pdf
Wisconsin	8/1	https://evers.wi.gov/Documents/COVID19/EmO01-FaceCoverings.pdf
Vermont	8/1	https://governor.vermont.gov/sites/scott/files/documents/ADDENDUM%202%20TO%20AMENDED%20AND%20RESTATED%20EXECUTIVE%20ORDER%20NO.%2001-20.pdf
