# Supplementary material for: #MaskOn! #MaskOff! Digital polarization of mask-wearing in the United States during COVID-19
Source: PLoS One. 2021 Apr 28;16(4):e0250817. doi: 10.1371/journal.pone.0250817 (PMC8081244; doi:10.1371/journal.pone.0250817)
Supplement: S3 Table — (PDF) [file pone.0250817.s006.pdf]

S3 Table. News Headlines of high-profile events between March and August 2020

Date	Media	News Headline	Category
3/1	Star Tribune (Minneapolis, MN)	3M can't confirm Pence comments about making more masks	N/A
3/4	Situation Room (CNN)	Pence: No need for Americans to buy masks	N/A
3/11	Washington Post	Face mask shortage propels CDC to loosen guidance for health-care workers	N/A
3/19	Situation Room (CNN)	CDC recommends health care workers reuse masks in a crisis and use bandanas or scarves as a last resort	N/A
3/21	Washington Post	Front-line health workers push back against the CDC's new mask guidelines	N/A
3/31	Washington Post	CDC considers advising the public to wear face coverings	N/A
4/3	CBS Evening News with Katie Couric	CDC recommends Americans should wear masks in public	CDC guidelines
4/3	Situation Room (CNN)	CDC recommends voluntary use of non-surgical masks	CDC guidelines
4/3	Philadelphia Inquirer, The (PA)	People should wear a cloth mask or facial covering when in public, new CDC guidance to say	CDC guidelines
4/4	Daily Mail	Trump: I call on every American to wear a face mask... except me.	Mixed messaging
4/4	New York Times	Undercutting C.D.C., President says he won't wear mask. (cover story)	Violation of CDC guidelines
4/4	Washington Post	New face mask guidance comes after battle between White House and CDC	CDC guidelines
4/5	Situation Room (CNN)	Trump on masks: I would wear one if I thought it was important	Violation of CDC guidelines
4/6	Daily Mail	Vain Trump lets the mask slip.	Violation of CDC guidelines
4/14	Washington Post	Without a mask, Trump shows us his true face	Violation of CDC guidelines
4/28	Situation Room (CNN)	V.P. Pence tours mayo clinic without a mask despite policy requiring visitors to wear them	Violation of CDC guidelines
4/28	Star Tribune (Minneapolis, MN)	No face mask for Mike Pence as he tours Mayo Clinic's COVID-19 testing labs	Violation of CDC guidelines
4/28	Washington Post	Pence visits Mayo Clinic without wearing a mask	Violation of CDC guidelines

4/29	New York Times	Pence flouts face mask rule in Mayo Clinic visit	Violation of CDC guidelines
4/30	Star Tribune (Minneapolis, MN)	Pence should have worn a mask during visit, and Mayo should have insisted	Violation of CDC guidelines
5/1	New York Times	Pence wears mask, a day after a visit without one	Compliance with CDC guidelines
5/2	Washington Post	Pence's staff targets reporter over tweet on masks	Violation of CDC guidelines
5/6	Daily Mail	No mask, but Trump goes for goggles.	Violation of CDC guidelines
5/11	CBS Evening News with Katie Couric	White House directs staff to wear face masks in the West Wing	Compliance with CDC guidelines
5/12	Evening Standard	White House staff must wear masks … except for 'invulnerable' Trump.	Mixed messaging
5/12	Daily Mail	White House to wear masks... except Trump.	Mixed messaging
5/12	Washington Post	White House mandates masks, but not for Trump	Mixed messaging
5/21	CBS Evening News with Katie Couric	President Trump visits Ford factory without face mask despite company policy	Violation of CDC guidelines
5/21	Situation Room (CNN)	Trump continues refusal to wear mask in public	Violation of CDC guidelines
5/21	Washington Post	White House mandates masks, but not for Trump	Mixed messaging
5/22	Evening Standard	Trump faces flak for taking off face mask during public part of Ford tour	Violation of CDC guidelines
5/22	Washington Post	Trump skips mask in plant visit, defying Ford's request and Michigan law	Violation of CDC guidelines
5/23	Daily Mail	Trump won't wear mask in public.	Violation of CDC guidelines
5/23	Daily Mail	At last, Trump wears mask (sort of).	Compliance with CDC guidelines
5/24	CNN Reliable Sources	Trump on his refusal to keep wearing mask at ford plant: I didn't want to give the press the pleasure of seeing it	Violation of CDC guidelines
5/26	Evening Standard	Biden is mocked by Trump after he wears face mask at Memorial Day event.	Violation of CDC guidelines
5/27	CBS Evening News with Katie Couric	Trump mocks those wearing face masks, calling it "politically correct"	Violation of CDC guidelines
5/30	Dayton Daily News (OH)	CDC publishes guide for schools on masks, cafeteria, buses, distancing	CDC guidelines
6/13	Washington Post	CDC encourages masks for rallies	CDC guidelines

6/13	Wall Street Journal - Online Edition	CDC encourages wearing masks, other coronavirus precautions at gatherings.	CDC guidelines
6/28	Situation Room (CNN)	Pence wears a mask during Texas visit as U.S. cases surge	Compliance with CDC guidelines
6/28	Situation Room (CNN)	Vice President Mike Pence urges everyone to wear a mask	Compliance with CDC guidelines
6/29	Washington Post	Pence urges mask-wearing as cases soar	Compliance with CDC guidelines
6/30	Situation Room (CNN)	CDC pleads with young people to wear masks	CDC guidelines
7/1	CBS Evening News with Katie Couric	President Trump says "masks are good," but does not support making them mandatory	Mixed messaging
7/1	Situation Room (CNN)	Trump: "All for masks," Don't need to be mandatory	Mixed messaging
7/2	Evening Standard	Trump: I'm all for face masks. I look like Lone Ranger in mine.	Compliance with CDC guidelines
7/10	Situation Room (CNN)	Trump refuses to wear mask publicly in virus hot spot	Violation of CDC guidelines
7/11	Situation Room (CNN)	Trump wears face mask while visiting wounded U.S. troops	Compliance with CDC guidelines
7/11	Situation Room (CNN)	Trump wears face mask on visit to Walter Reed Medical Center	Compliance with CDC guidelines
7/12	Weekend Edition Sunday (NPR).	Politics chat: President Trump changes stance on masks, wears one in public.	Compliance with CDC guidelines
7/12	Good Morning America (ABC)	President tours military hospital in mask.	Compliance with CDC guidelines
7/12	New York Times	At a hospital, Trump finally dons a mask.	Compliance with CDC guidelines
7/13	Good Morning America (ABC)	Trump wears mask in public	Compliance with CDC guidelines
7/13	Washington Post	In about-face, Trump dons mask at Walter Reed	Compliance with CDC guidelines
7/17	Situation Room (CNN)	Trump: Masks cause problems too	Violation of CDC guidelines
7/18	Situation Room (CNN)	Trump cites freedom as reason to not mandate masks	Violation of CDC guidelines
7/20	FOX News	Trump tweets photo of himself wearing coronavirus mask: ‘nobody more Patriotic than me’	Compliance with CDC guidelines
7/20	Situation Room (CNN)	Trump posts picture in mask	Compliance with CDC guidelines

7/20	CBS Evening News with Katie Couric	President Trump briefings to return as he calls wearing a mask patriotic	Compliance with CDC guidelines
7/21	Situation Room (CNN)	Trump says he would wear a mask, but did not last night at Trump Hotel	Violation of CDC guidelines
7/31	Situation Room (CNN)	Trump in Florida as State sees deadliest day of pandemic, greets crowds without mask	Violation of CDC guidelines
