# Supplementary material for: #MaskOn! #MaskOff! Digital polarization of mask-wearing in the United States during COVID-19
Source: PLoS One. 2021 Apr 28;16(4):e0250817. doi: 10.1371/journal.pone.0250817 (PMC8081244; doi:10.1371/journal.pone.0250817)
Supplement: Attachment — Submitted filename: PLSONE_responses to reviewer comments.docx [file pone.0250817.s007.docx]

PONE-D-21-03538
#MaskOn! #MaskOff! Digital polarization of mask-wearing in the United States during COVID-19

The authors appreciate the opportunity to submit a revised draft of the manuscript “#MaskOn! #MaskOff! Digital polarization of mask-wearing in the United States during COVID-19” for publication in PLOS ONE. We appreciate the time and effort that the academic editor and the four reviewers dedicated to providing comments and suggestions, which have helped us implement valuable improvements to our paper. We have addressed all the journal requirements and incorporated most of the suggestions made by the reviewers in the revised paper, detailed below in point-by-point responses. We have attached the revised paper with changes highlighted in addition to a “clean” copy. We will be willing to make further revisions if necessary.

Journal Requirements:

1. Please ensure that your manuscript meets PLOS ONE's style requirements, including those for file naming. The PLOS ONE style templates can be found at

[https://journals.plos.org/plosone/s/file?id=wjVg/PLOSOne_formatting_sample_main_body.pdf](https://urldefense.com/v3/__https:/journals.plos.org/plosone/s/file?id=wjVg*PLOSOne_formatting_sample_main_body.pdf__;Lw!!C5qS4YX3!WBzHJNtMQHLiVPZTve5EBcO1tMXKL5HOS2xJgNM6e1qTRcbDFPRRCRvHoqWUB80r30M$) and

[https://journals.plos.org/plosone/s/file?id=ba62/PLOSOne_formatting_sample_title_authors_affiliations.pdf](about:blank)

Author response:

We have formatted the revised paper following PLOS ONE style requirements, using the PLOS ONE style templates.

2. PLOS ONE has specific requirements for studies using personal data from third-party sources, including social media, blogs, other internet sources, and phone companies ([https://journals.plos.org/plosone/s/submission-guidelines#loc-personal-data-from-third-party-sources](https://urldefense.com/v3/__https:/journals.plos.org/plosone/s/submission-guidelines*loc-personal-data-from-third-party-sources__;Iw!!C5qS4YX3!WBzHJNtMQHLiVPZTve5EBcO1tMXKL5HOS2xJgNM6e1qTRcbDFPRRCRvHoqWURoeOIno$)). These requirements include confirming data are collected and used in accordance with the company or website’s Terms and Conditions, obtaining appropriate ethics or data protection body review, and ensuring appropriate consent from individuals whose data are used in research. In this case, please ensure that your Ethics statement is in compliance with guidelines, and that you have complied with the company's (i.e., Twitter's) Terms and Conditions, with appropriate permissions.

Author response:

Our data was collected and used according to Twitter’s Terms and Conditions. This study has been reviewed by the University of Oregon Research Compliance Services (RCS) under the 2018 Common Rule and determined to qualify for exemption under Title 45 CFR 46.104(d)(4).

3. We note that you have stated that you will provide repository information for your data at acceptance. Should your manuscript be accepted for publication, we will hold it until you provide the relevant accession numbers or DOIs necessary to access your data. If you wish to make changes to your Data Availability statement, please describe these changes in your cover letter and we will update your Data Availability statement to reflect the information you provide.

Author response:

Below is our revised Data Availability statement--

We provided the search criteria used to query the API and retrieve the hashtag data in the SI. This will allow others to access the same data analyzed in this study. The data are available for viewing on the OSF data repository site using the following link:

<https://osf.io/vgcrf/?view_only=1163e1cad81847f9a63115cae8da46e2>

The data are also available from the corresponding author upon reasonable request and subject to Twitter’s Terms and Conditions governing the sharing of Twitter data. 

[Note: HTML markup is below. Please do not edit.]

Reviewers' comments:

Reviewer's Responses to Questions

**Comments to the Author**

1. Is the manuscript technically sound, and do the data support the conclusions?

The manuscript must describe a technically sound piece of scientific research with data that supports the conclusions. Experiments must have been conducted rigorously, with appropriate controls, replication, and sample sizes. The conclusions must be drawn appropriately based on the data presented.

Reviewer #1: Partly

Reviewer #2: Partly

Reviewer #3: Yes

Reviewer #4: Yes

2. Has the statistical analysis been performed appropriately and rigorously?

Reviewer #1: Yes

Reviewer #2: Yes

Reviewer #3: I Don't Know

Reviewer #4: Yes

3. Have the authors made all data underlying the findings in their manuscript fully available?

The [PLOS Data policy](https://urldefense.com/v3/__http:/www.plosone.org/static/policies.action*sharing__;Iw!!C5qS4YX3!WBzHJNtMQHLiVPZTve5EBcO1tMXKL5HOS2xJgNM6e1qTRcbDFPRRCRvHoqWUhx5hHEA$) requires authors to make all data underlying the findings described in their manuscript fully available without restriction, with rare exception (please refer to the Data Availability Statement in the manuscript PDF file). The data should be provided as part of the manuscript or its supporting information, or deposited to a public repository. For example, in addition to summary statistics, the data points behind means, medians and variance measures should be available. If there are restrictions on publicly sharing data—e.g. participant privacy or use of data from a third party—those must be specified.

Reviewer #1: Yes

Reviewer #2: No

Reviewer #3: Yes

Reviewer #4: Yes

4. Is the manuscript presented in an intelligible fashion and written in standard English?

PLOS ONE does not copyedit accepted manuscripts, so the language in submitted articles must be clear, correct, and unambiguous. Any typographical or grammatical errors should be corrected at revision, so please note any specific errors here.

Reviewer #1: Yes

Reviewer #2: Yes

Reviewer #3: Yes

Reviewer #4: Yes

5. Review Comments to the Author

Please use the space provided to explain your answers to the questions above. You may also include additional comments for the author, including concerns about dual publication, research ethics, or publication ethics. (Please upload your review as an attachment if it exceeds 20,000 characters)

Reviewer #1: Thank you for the opportunity to review “#MaskOn! #MaskOff! Digital polarization of mask-wearing in the United States duringCOVID-19.” This is an interesting study of an obviously important and ongoing subject. At this point, I cannot recommend publication, as I believe the manuscript is too disconnected from some important political phenomena. However, I do hope to see a version of this in print, and I have a number of suggestions below that I hope will help.

My main concern about this manuscript is its disconnection from politics. That is, the authors chart some interesting trends in Twitter use of various mask-related hashtags, but it’s not clear *why* we see the various peaks and valleys in the data, or whether the Twitter patterns are instigating or following other discussions in society. The authors do attempt to correlate these tweet trends with some media headlines, but these prove insufficient. For example, is the dip in hashtag use in early June and then the sudden resurgence a few weeks later related to Covid disease rates? Does it track media coverage of the illness? Is it a function of the George Floyd protests of early June that temporarily dominated the national conversation? More generally, do disease rates or media trends track hashtag usage across the larger time span?

Author response:

The reviewer made an important point about connecting this study to politics, COVID-19 disease rates, and media trends in the time span of interest. Based on the reviewer’s suggestion, we have revised the paper in the following ways.

1) We have included in the analysis COVID-19 disease trends. We describe the data source on page 5, the methodology on page 7, and report the results of the analysis on page 12, which showed strong positive correlations between daily confirmed coronavirus cases and both types of hashtags. We added Fig 7 (p. 12) to visualize the relationships. In the Discussion section, on page 13, we discuss these correlations in terms of how uncertainties about the impacts of the pandemic grew in response to the surges of the disease case counts, linking the hashtag trends to the disease trends.
2) We agree with the reviewer that our previous analysis did not sufficiently explore the news trends. In revising the paper, we annotated and classified a total of 62 mask-related U.S. news headlines identified in the period of interest (see p. 6 for the description of the annotation and classification of the headlines, and see the S3 Table in SI for all the headlines included in the analysis). In the Results section, we plotted the distributions of news categories along the temporal trends of pro-mask hashtags in Fig 1 on page 10, and long the temporal trends of anti-mask hashtags in Fig 2 on page 10, and in Fig 3 where the trends of the top three pro- and anti-mask hashtags are plotted together in a stacked area graph. These plots show that the clusters of news headlines are roughly concurrent with the hashtag peaks, especially in the early months of the pandemic. In the Discussion section, on page 13, we contextualize the hashtag trends in light of the clusters of news headlines, drawing inference about the role of the news coverage in magnifying the rhetorical polarization on mask wearing. We also discuss how the rhetorical polarization was exacerbated by the information crisis that accompanied the pandemic.
3) To further contextualize the hashtag trends in a big picture of the relevant events related to COVID-19, we added on page 12 Fig 8 showing side by side (A) daily trends of the hashtags alongside COVID-19 disease case counts and (B) monthly trends of three circumstantial data: statewide mask mandates, news headlines, and Google search interests. We discuss these trends on page 15, noting that the converging downturns in early June indicate that collective attention was directed away from the pandemic at the time of reopening that coincided with the nationwide protests against police brutality set off by the murder of George Floyd.

I was also struck by how many more pro-mask tweets there were than anti-mask tweets. As the authors note, this is somewhat consistent with public opinion and public behavior, and Democrats are somewhat more prominent on Twitter than Republicans. However, what is striking to me is how much attention anti-maskers received relative to their numbers. (This might again be an area to bring in a media analysis.) Perhaps this is a function of their controversial nature, which makes for good news stories, or their association with violent activism in state legislatures, or that these messages were largely echoed by the President. But by my read, the most prominent pro-mask tweets outnumbered the most prominent anti-mask tweets by a factor of ten, making it unclear why or how this was even considered a debate. I’m assuming this is an accurate depiction of Twitter activity on masking, although it makes me curious whether anti-maskers use hashtags not detected by the authors or perhaps eschew hashtags altogether. Is it possible to examine tweets in another way looking for content other than hashtags on this topic?

Related to this, I was wondering if anti-maskers had used Twitter to elevate their prominence in excess of their numbers in the population and draw attention to their efforts, or if they were essentially out-gunned on Twitter, relying on other political methods to capture attention.

Author response:

The reviewer rightly pointed out that the two sides of the polarization are exceedingly unbalanced in terms of the volume of the opposing hashtags, so much so that one wonders if the situation should even be considered a debate. Our response to this question is part of a larger revision that we have undertaken to address political polarization in light on the relevant research. Specifically, we conducted a focused review of the literature on political participation in relation to political polarization. Through the literature review we came to realize that it is not only empirically important, but also theoretically significant to view political polarization as both a rhetorical phenomenon and as a participatory process. This is the central framework within which we formulate and test hypotheses in the revised paper. The results answer the reviewer’s question about the imbalance between the two sides of the debate, and more generally, the revisions address the concern over our theoretical framework raised by reviewer #2. Details are as follows.

The focused literature review on political polarization (see page 2, para 2,3,4) provided an empirical ground for distinguishing rhetorical polarization and participatory polarization as two levels of political polarization. Based on existing evidence and the issue-specific context of COVID-19, we formulated three hypothesis about the digital polarization over mask wearing on Twitter: 1) strong rhetorical polarization 2) uneven participatory polarization in favor of a pro-mask majority and 3) echo chamber effect in the majority group.

Our qualitative content analysis of the 35 hashtags shows an emotionally charged and clearly delineated rhetorical polarization, which escalated over time, as seen in the parallel exponential growths of both groups of hashtags. Our quantitative analysis of the volumes of the two groups of hashtags shows an extremely lopsided polarization as a participatory process whereby mask supporters outgunned mask resistors and stayed in echo chambers insulated from mask resistors. We discuss the participatory imbalance on page 14 in light of the type-token distributions of the hashtags and the comparison between our data and the Knight Foundation and Pew Research data. Based on our results, we argued that the skewness in participation suggests Americans stepped up in strong support for masking despite the strongly polarizing rhetoric about the policy and the news reports that magnified the rhetorical polarization.

As stated above, based on the reviewer’s suggestion of a media analysis, we annotated 62 news headlines about events related to the CDC mask recommendation and its enforcement by the Trump administration. We plotted the temporal distributions of the headlines together with the temporal trends of the hashtags to contextualize the digital discourse about masking in Figs 1,2,3 on page 10, showing that major peaks in hashtag volume roughly tracked the distributions of news clusters over the time span. In the Discussion, on page 13, we discuss the role of the media in exacerbating the rhetorical polarization by magnifying the anti-mask rhetoric, as seen in the clusters of news reports on White House violations of CDC mask guidelines, especially in the early days of the pandemic. We added citations on media bias and cognitive bias that underlie the greater attention paid to the negative news.

To the reviewer’s question whether some anti-mask hashtags were not detected in the study, we would like to respond by noting that the query method we used was the same for both types of hashtags. Should the method miss any relevant hashtags, the problem would likely impact the retrieval of both types of hashtags. As to whether users eschewed hashtags altogether and used non-hashtag contents, this possibility could not be ruled out. On page 4, we have added language to explain our rationale for focusing on hashtags in this study. On page 15, we stated the potential limitations of this research focus.

The authors include some commentary in their discussion section noting the patterns of discussion across mask networks, with mask tweeters mostly tweeting among their own groups, but occasionally engaging those of the opposing viewpoint. The authors suggest, rather fatalistically, that “echo chambers” can induce polarization, but that engagement with people of opposing viewpoints can also induce polarization. Yet surely there are forms of interaction that can depolarize communities. Twitter might not be the best venue for such interaction, but surely some of that occurred. I would encourage the authors to investigate the political communications or political networks literature on this subject.

Author response:

We appreciate the reviewer’s suggestion, which converges with the suggestion by Reviewer #2. In response to these helpful suggestions, we reviewed the literature on echo chambers in relation to political polarization. On page 2 we summarize the literature, addressing two important themes that emerged from the literature. These contribute to our conceptualization of a theoretical framework: 1) polarization is issue-specific whereby rhetorical polarization does not translate to equal participation divided along partisan lines, 2) value-aligned exchanges in echo chambers overwhelmingly outnumber cross-ideological interactions, suggesting strong imbalance in participatory polarization.

The conceptual distinction between the two levels of polarization allowed us to characterize the findings more accurately: there was a sharp rhetorical polarization on the one hand, and there was a lopsided participatory polarization with the majority group in an echo chamber, on the other hand.

On page 15, para 1, in light of the improved characterization of our findings, we revised our previous general statement about exposure to opposing views increasing polarization (e.g. Bail et al. 2018). The revision specifically addresses what this kind of exposure looks like in light of our findings: Twitter users are more likely to be exposed to pro-mask views than anti-mask views due to the absolute dominance of the former. Thus, the backfire effects found in Bail et al. (2018) are more likely to exacerbate the mask resistors and make them more committed to their resistance.

We point out the false equivalence often associated with opposing views when the fringe group is given attention disproportionate to their size due to media bias, as noted by the Reviewer, and the general bias in affective information processing whereby people simply pay more attention to negative events. Citations on this have been added.

Related to that topic, I found Figure 4 rather difficult to interpret, and the accompanying text was not terribly Illuminating.

Author response:

We have revised the caption and description of Fig 4, which is now Fig 5 in the revised paper, on page 11. We added language that clarifies the contrastive distributions of the two groups of tweeters with a focus on the 0-reply bar, which shows the proportion of that part of each group that pays zero attention to their opponents in their replies. The taller the 0-reply bar, the higher the level of intragroup interaction. We hope the improved caption can reduce the difficulty interpreting the plot.

In general, I encourage the authors to situate this research more in the political phenomena surrounding the Twitter usage, rather than just the Twitter usage itself.

I hope these comments are useful.

Author response:

All the comments were useful to us in improving our paper and are greatly appreciated. The reviewer’s suggestion that we situate this research more in the political phenomena surrounding the hashtag usage has been well taken, and we believe we have successfully incorporated the suggestion in the revised paper. The revisions enable us to contribute to the debate on political polarization in an important way.

Reviewer #2: In this paper the authors analyze 412,959 stance-bearing hashtags about mask wearing and identify 35 unique hashtags related to mask wearing. Ultimately, they argue that the use of these hashtags exhibit typical signs of modern partisan polarization within American politics, including conflict that invokes emotional responses and the siloing of ideas into echo chambers. This paper provides a unique approach and a relatively robust look at some interesting data. However, the manuscript is not prepared for publication. The authors lack a clear and articulated theoretical underpinning for their findings that might drive hypothesis. Ultimately, the authors bury their lead at the end and need to engage in significant revision to unpack their claims and the theory they are building upon for this manuscript to be publishable.

Author response:

We greatly appreciate the reviewer’s recognition of the strengths and potentials of our study. The reviewer’s comments on the weaknesses of our study, especially the glaring omission of a theoretical framework, have helped us make substantial improvements, detailed below in point-to-point responses.

I have included a number of notes below. In the interest of illustrating the main issue, that you bury a lead which is actually quite compelling, I preserved all my comments as I wrote them so that you can follow the train of thought a reader might go through.

First, I would like to know more about your qualitative coding scheme for the 35 hashtags, including how many coders participated, was there intercoder reliability across the tests, and finally how did you deal with tags that invoked multiple categories. In addition, was the content of the tweet analyzed in any way to confirm that the hashtags are being used legitimately? In other words, could people sarcastically use the hashtag #maskskill in order to undermine that campaign. I am also interested in your use of the #coverup hashtag. Is that not a mask hashtag? Consider you are writing for a broad audience at PlosOne you really need to unpack this more.

Author response:

We added on pages 5-6 in the Methodology section (under Qualitative analysis) a description of the coding and classification of the 35 hashtags with information on intercoder reliability.

In response to the question about how to handle hashtags that invoked multiple categories, we clarified that the subcategorization was based on a granular semantic analysis based on the lexical and grammatical resemblances between the hashtags and that we recognize that subcategories thus identified have fuzzy boundaries while they differ in semantic focus in important ways.

In response to the question about the context in which hashtags are used, we added language and citations on page 4 in the last para of the Introduction, stating the unique meme-like characteristics of hashtags that allow them to “take on a life of their own through repeated sharing in largely unchanged forms, and as such play a vital role in viral trending of ideas”.

We hope that this statement helps make the point that, although we could not rule out sarcastic uses of hashtags in certain contexts, such contexts ultimately do not matter because hashtags are by design both structurally independent and functionally unique as topical markers of the central ideas of tweets.

We reviewed our data on #coverup and decided that it is both unnecessary as a control and potentially confusing due to its occasional uses in the literal sense that is mask-related. So we removed it from the analysis.

Your discussion of the text content of mask tags is striking, though I am surprised that you include merely the N for the number of tags that, say, employ expletive attributes. I would be much more interested in a clearer discussion of the frequency in use of those attributes relative to other attributes. The existence of these tags is less interesting, their use is far more interesting and speaks volumes to the narrative around mask wearing. The paragraph from lines 162-172 could be divided into two and include this additional discussion of relative frequency of use.

Author response:

We followed the reviewer’s suggestion and calculated the relative frequency of the hashtags with the expletive attributives in comparison to other pro-mask but non-expletive hashtags. The data is provided in the newly added Table 3 on page 9, and the results are reported on pages 8-9.

Having arrived at your results section, I am struck by your lack of clear theory and supported hypotheses. I really think there is room before the methodology section to spend more time discussing the current literature on polarization and social media interaction to make your claims more clear.

Author response:

We are grateful for this suggestion. See below for details on the revisions.

The paragraph from lines 194-210 is really interesting and compelling with regard to the literature on group-think and echo chambers within social media platforms. This might be a way to incorporate more theoretical grounding within the manuscript. This improved ground could even generate some interesting testable hypothesis regarding the use of tags to 1.) signal identity vs 2.) convince opposition (which implies interaction). Lines 216-228 further emphasize this need for theoretical underpinnings to the argument.

On page 2 in the Introduction, we have added two paragraphs that unpack the literature on political polarization and echo chambers. In para 2, we reviewed four recent studies on political polarization that converge on one important finding. That is, the echo chamber effect tends to be observed in the larger and more dominant group of a debate. This suggests to us the need to distinguish polarization as a rhetorical phenomenon and polarization as a participatory process. In para 3, we discuss the kind of issues covered in the literature on political polarization and extracted evidence from this body of literature that political polarization is issue-specific.

On pages 2-3, we discuss further evidence that political polarization is not only issue-specific, but that the binary distinction between political issues and non-political issues is inadequate and cannot capture the gradience between the various issues studied in the literature, and does not account for polarization as a dynamic participatory process.

This literature review turned out fruitful and theoretically rewarding in that it generated a theoretical perspective from which we can critically examine political polarization at two related, but distinguishable, levels. Based on the insights gained from the literature review along with the four contextual forces surrounding the COVID-19 pandemic that we have identified, we formulated three testable hypotheses: 1) Twitter discourse on mask wearing is rhetorically polarized, 2) participation in the rhetorical polarization is asymmetric in favor of a pro-mask majority and 3) the echo chamber effect is more likely in the pro-mask majority group than in the anti-mask minority group.

See discussion on pages 12-15 on how the results confirm our hypotheses, as well as the surprise finding that as a participatory process, the digital polarization of mask wearing was even more lopsided than would have been predicted by the Twitter baseline ideology distributions and the issue-based ideology distributions, but was highly consistent with the national survey on mask wearing that showed broad support for masking. The findings demonstrate that a rhetorical polarization, no matter how uncivil, is not necessarily matched by a participatory process equally divided along partisan lines. The findings caution against the false equivalence often associated with opposing stances when the fringe group is given attention disproportionate to its size, mostly by the media that is generally biased toward bad news. The revised study makes a theoretical contribution to the debate on political polarization.

Ultimately, your argument really hits its stride with the first paragraph of the discussion section. Unfortunately, you have buried the lead here. This needs to be up front, with some theory behind it and some expectations in the form of hypotheses. You pack a lot of citations into this paragraph (for example, line 293). These need to be unpacked in a more robust way that produces hypotheses to test.

Author response: We agree and appreciate the comment. See the response above.

The paragraph from line 271-283 seems like additional analysis in the discussion section. It seems disjointed placed there. Perhaps it would better fit in the results section as an alternative explanation or as a potential explanation of interaction. What might be interesting to see is if major events, like CDC announcements or COVID increases produced actual conflict as opposed to mere digital echo chambers. This would make better use of this data and add a layer to your analysis.

Author response:

We have moved this part to the Results section (pages 10-11). In the discussion session, we added language addressing this finding on page 13 in the discussion.

In terms of your main argument, I think a figure plotting the top 3 pro and anti-mask figures over time would be really interesting. Again, I think you need a theoretical hook here. Perhaps identifying events which drive interaction between the two echo chambers might be an interesting direction. Splitting your Fig 1 and Fig 2 into separate figures makes this less clear.

Author response:

We have added on page 10 Fig 3 plotting the top 3 pro- and anti-mask hashtags in a stacked area graph that shows clearly the volumes of the pro-mask hashtags overshadow their anti-mask counterparts in extremely unbalanced proportions over the period of interest. This provides visual illustration of the evidence in support of our hypothesis about the uneven participation of the two sides.

We have kept Fig 1 and Fig 2 for the purpose of showing the respective temporal trends with greater detail and added news headline clusters that indicate events that concurred with the temporal peaks in the hashtag trends.

Other significant events have been added as well.

Fig 7 shows how the hashtag trends correlated with the COVID-19 disease trends.

Fig 8. shows additional circumstantial data including the temporal trends of the monthly data on statewide mask mandates, news headlines, and Google search trends for “face mask” side by side with the daily counts of both coronavirus case counts and hashtag counts. We interpret the converging trends in the discussion on page 13 in terms of how public attention to the pandemic shifted over time, the factors that potentially contributed to the shift, and how the attention trajectories help contextualize the hashtag trends.

Ultimately, to reiterate, this paper presents a really interesting analysis without providing any compelling theoretical hook up front to draw the reader in. Further, when you come around to your theoretical claim, you pay passing reference without unpacking the implications of your discoveries on the broader literature on polarization and social media use. This is the most glaring omission in this paper and needs to be corrected before publication.

Author response:

As detailed above, we have addressed the theoretical oversight in the revisions.

Other editorial notes:

Table 1: The top line repeats twice.

Author response: This has been fixed.

Equation 4: Why? Most of us know what averages are. This seems excessive.

Author response: The equation was intended to provide a clear definition of Bias as we use it in our analysis.

The resolution on all of your figures is really poor. Be sure this gets resolved if you make it to the proofing phase.

Author response: we have reformatted all the figures using the Preflight Analysis and Conversion Engine (PACE) digital diagnostic tool to ensure correct formatting and high resolution.

Reviewer #3: The study seems to be well-constructed and carried out. I would not require changes but will point out two possible missed opportunities, which the authors might consider adding at their discretion, depending on how data has been collected and stored. The first is sentiment analysis for the tweets using positive and negative mask hashtags—this seems significant and would add a noteworthy dimension to the data which could not account for other significant demographic vectors (314, 325-30). The second is that the "echo chamber" effect might be further illuminated beyond "interactions between the tweeters" (291) if it were possible to plot, using social network analysis, the follower and mutual followers among and between the hashtag-using communities. I imagine that, if significant enough to be valuable, these might help to describe more fully the behaviors of distinct groups (such as mask makers, trolls, potentially even bot behaviors). Both introduce substantial complexity, but, I believe, potential value added for the study, again, at the authors' discretion.

Author response:

We appreciate the author’s positive feedback on our study and we recognize that our study has limitations as pointed out by the reviewer.

We added a statement on p. 4 defining hashtags and explaining why we focused on them in this study. We added a paragraph at the end of the Discussion section on page 15 where we state the limitations of the study, including the two pointed out by the reviewer: the lack of sentiment analysis of larger tweets, and the lack of a social network analysis, which would have provided a fuller picture of the stance distributions and the interaction between the users.

Reviewer #4: The article addresses an interesting topic regarding the politics of mask wearing during the pandemic. They conduct this analysis by scraping Twitter to find useage of various hashtags that are related to both pro- and anti-mask wearing. The idea and execution of this analysis is well done. I recommend it be published.

The authors appreciate the positive feedback.

6. PLOS authors have the option to publish the peer review history of their article ([what does this mean?](https://urldefense.com/v3/__https:/journals.plos.org/plosone/s/editorial-and-peer-review-process*loc-peer-review-history__;Iw!!C5qS4YX3!WBzHJNtMQHLiVPZTve5EBcO1tMXKL5HOS2xJgNM6e1qTRcbDFPRRCRvHoqWUTulhEIU$)). If published, this will include your full peer review and any attached files.


If you choose “no”, your identity will remain anonymous but your review may still be made public.


**Do you want your identity to be public for this peer review?** For information about this choice, including consent withdrawal, please see our [Privacy Policy](https://urldefense.com/v3/__https:/www.plos.org/privacy-policy__;!!C5qS4YX3!WBzHJNtMQHLiVPZTve5EBcO1tMXKL5HOS2xJgNM6e1qTRcbDFPRRCRvHoqWU9-tGFaA$).

Reviewer #1: No

Reviewer #2: No

Reviewer #3: No

Reviewer #4: No

[NOTE: If reviewer comments were submitted as an attachment file, they will be attached to this email and accessible via the submission site. Please log into your account, locate the manuscript record, and check for the action link "View Attachments". If this link does not appear, there are no attachment files.]

While revising your submission, please upload your figure files to the Preflight Analysis and Conversion Engine (PACE) digital diagnostic tool, [https://pacev2.apexcovantage.com/](https://urldefense.com/v3/__https:/pacev2.apexcovantage.com/__;!!C5qS4YX3!WBzHJNtMQHLiVPZTve5EBcO1tMXKL5HOS2xJgNM6e1qTRcbDFPRRCRvHoqWUoPNcFMI$). PACE helps ensure that figures meet PLOS requirements. To use PACE, you must first register as a user. Registration is free. Then, login and navigate to the UPLOAD tab, where you will find detailed instructions on how to use the tool. If you encounter any issues or have any questions when using PACE, please email PLOS at [figures@plos.org](mailto:figures@plos.org). Please note that Supporting Information files do not need this step.
